# The most common errors in the management of gastroesophageal reflux

**DOI:** 10.1186/1824-7288-41-S2-A32

**Published:** 2015-09-30

**Authors:** Ruggiero Francavilla, Cinzia Ciullo, Claudio Cafagno

**Affiliations:** 1 Ospedale Pediatrico Giovanni XXIII – Università degli studi di Bari, Italy

## 

Gastro-Esophageal Reflux (GER) occurs in more than two-thirds of otherwise healthy children and is one of the major reason for referral to paediatricians; in fact, a quarter of all paediatric visits in the first six months of life [1] and numerous accesses to the paediatric gastroenterologist services are secondary to GER. GER is defined as the passage of gastric contents into the esophagus and is different from gastroesophageal reflux disease (GERD), which includes all the symptoms and/or complications associated with GER [2].

GER is considered a physiological normal process which occurs several times a day in healthy infants, children and adults. GER is usually associated with transient lower esophageal sphincter relaxations independent from swallowing, which allow the passage of gastric contents into the esophagus. Episodes of GER in healthy adults tend to occur after meals, lasting less than three minutes and usually do not cause symptoms. Although we know less on the normal physiology of newborns and infants, regurgitation is the most visible symptom and tends to occur daily in 50% of all infants with a peak incidence between 4 and 6 months of life [3].

For the majority of paediatric patients (especially in infants), the clinical history and the objective appraisal, in the absence of danger signals, are sufficient to reliably diagnose a not complicated GER and initiate conservative treatment strategies; in general, the diagnostic tests are not always necessary. The reliability of the symptoms to make the clinical diagnosis of GERD is higher in children younger than 8 years, reporting heartburn: only in this case, the doctor can make a diagnosis of Gastro Esophageal Reflux Syndrome and give an indication for therapy [4]. To date, no single symptom or set of symptoms can be trusted, and then used to diagnose GERD in children or to predict which patients are more likely to respond to therapy [2]. The only exception is the child (age> 8y.o.) reporting a history of long-term heartburn with or without vomiting.

The new ESPGHAN/NASPGHAN guidelines [2] describe different treatment options for the treatment of infants / children with GER and GERD. In particular, they emphasized the changes in lifestyle, because it can effectively reduce the symptoms of both infants and children. They are based on a combination of changes of milk formulas and positional therapy. In infants with GER, it can be effective the changes to the mother's diet if children are breastfed, change the formula in use, reducing the volume of the single meal associated with an increased frequency of feedings. In particular, the guidelines stress that the allergy to milk proteins may have a clinical presentation similar to GERD. A strategy of nutritional intervention involves the use of thickened formulas that are able to decrease the regurgitation. A recent meta-analysis has shown that the anti regurgitation formula have the following functions: a) increase the number of infants without regurgitation; b) reduce the number of episodes of regurgitation and vomiting daily; c); increase the weight recovery of the infant, d) although they do not change the 24 hours pH study [4].
